# “My Patients Asked Me If I Owned a Fruit Stand in Town or Something.” Barriers and Facilitators of Personalized Dietary Advice Implemented in a Primary Care Setting

**DOI:** 10.3390/jpm11080747

**Published:** 2021-07-29

**Authors:** Heather L. Rogers, Silvia Núñez Fernández, Susana Pablo Hernando, Alvaro Sanchez, Carlos Martos, Maribel Moreno, Gonzalo Grandes

**Affiliations:** 1 Biocruces Bizkaia Health Research Institute, 48903 Barakaldo, Bizkaia, Basque Country, Spain; silay60@gmail.com; 2 Ikerbasque Basque Foundation for Science, 48009 Bilbao, Bizkaia, Basque Country, Spain; 3 Primary Care Research Unit of Bizkaia, Biocruces Bizkaia Health Research Institute, Osakidetza, 48903 Barakaldo, Bizkaia, Basque Country, Spain; a121944@usal.es (S.P.H.); Alvaro.SanchezPerez@osakidetza.eus (A.S.); GONZALO.GRANDESODRIOZOLA@osakidetza.eus (G.G.); 4 Arrigorriaga Health Center, Integrated Health Organization Barrualde-Galdakao, Osakidetza, 48480 Arrigorriaga, Bizkaia, Basque Country, Spain; CARLOS.MARTOSVALENCIA@osakidetza.eus; 5 Integrated Health Organization Barrualde-Galdakao, Osakidetza, 48960 Galdakao, Bizkaia, Basque Country, Spain; MARIAISABEL.MORENOLEAL@osakidetza.eus

**Keywords:** personalized dietary advice, lifestyle intervention, primary care, health promotion, disease prevention, behavior change intervention, implementation

## Abstract

Primary care is especially well positioned to address prevention of non-communicable diseases. However, implementation of health promotion activities such as personalized dietary advice is challenging. The study aim was to understand barriers and facilitators of the personalized dietary advice component of a lifestyle intervention in primary care, as perceived by health center professionals and program participants. Thirteen focus groups were conducted with 49 professionals and 47 participants. Audio recordings were transcribed. Professional group text was coded using the Consolidated Framework for Implementation Research (CFIR). Participant group text was coded via an inductive approach with thematic analysis. Across most CFIR domains, both barriers and facilitators were equally present, except for ‘characteristics of individuals’, which were primarily facilitators. Intervention characteristics was the most important domain, with barriers in design and packaging (e.g., the ICT tool) and complexity. Facilitators included high evidence strength and quality, adaptability, and relative advantage. Participants described the importance of more personalized advice, the value of follow-up with feedback, and the need to see outcomes. Both professionals and patients stated that primary care was the place for personalized dietary advice intervention, but that lack of time, workload, and training were barriers to effective implementation. Implementation strategies targeting these modifiable barriers could potentially increase intervention adoption and intervention effectiveness.

## 1. Introduction

Non-communicable diseases (NCDs) such as cardiovascular diseases, cancers, chronic respiratory diseases and diabetes are the leading cause of death in most countries worldwide [1]. These deaths are not limited to older age groups, as many occur in individuals 30 to 69 years of age and, when this occurs, are considered to be “premature”. Most of the premature morality due to NCDs can be avoided or delayed because modifiable behavioral risk factors such as unhealthy diet, lack of physical activity, tobacco use, and harmful alcohol use all increase the risk of NCDs [2]. The 2030 Agenda for Sustainable Development, adopted by all United Nation Member States in 2015, includes reducing premature mortality from NCDs by one-third by 2030 relative to 2015 as target 3.4 of its Sustainable Development Goals [3]. Yet, as of 2020, only approximately 15 countries are currently expected to meet this SDG target [4].

Healthy diets are an important component of primary and secondary prevention of NCDs. Population-level World Health Organization (WHO) recommendations for diet include achieving energy balance and a healthy weight; limiting energy intake from total fats; shifting fat consumption to unsaturated fats and away from saturated fats and transfatty acids; increasing consumption of fruits and vegetables, and legumes, whole grains and nuts; limiting free sugars intake; limiting salt (sodium) consumption; and consuming iodized salt [5]. For example, over 1.7 million deaths per year are attributed to diets lacking at least 400 g of fruits and vegetables a day [5]. Healthy diets with appropriate intake of fruits and vegetables can reduce the risk of certain NCDs, such as cardiovascular diseases [6], and show favorable effects on cardiovascular disease risk factors [7]. However, standard nutritional guidelines are typically based on population averages (e.g., European Food Safety Authority reference values [8]) and one-size-fit-all nutritional recommendations are not likely to be relevant to all individuals. One reason is because there are large differences in how individuals respond to different foods. One study of 1000 adults in the United Kingdom and the United States [9] suggests that there are large differences in metabolic responses between individuals, but an individual’s response to the same meal was often similar and predictable.

Therefore, personalized nutrition interventions have been considered to be a promising approach to improve dietary intake for health promotion and disease prevention. Although there is no agreed-upon definition, personalized nutrition or individually-tailored nutrition can refer to the use of information on individual characteristics to develop targeted nutritional advice, products, or services [10]. Individual characteristics refer to factors more specific than those available at the population level. Such factors may be (a) biological (e.g., blood biomarkers, differential response to foods or nutrients due to genotype or phenotype), (b) personal (e.g., current and past dietary behaviors, preferences, barriers, (c) intervention-related (e.g., objectives, intervention delivery), and/or (d) socio-environmental (e.g., factors that motivate and enable healthy eating patterns) [11]. One systematic review of 11 randomized controlled studies indicated that healthy adults receiving personalized nutrition advice improved their dietary intake to a greater extent than those receiving generalized dietary advice [12]. Cochrane reviews indicate that dietary advice can confer modest beneficial changes in diet and cardiovascular risk factors in the short term (less than one year), but longer-term effects are not known [13,14].

Lifestyle interventions, such as personalized nutrition or dietary advice interventions, can be carried out in many settings, including at work, in the community, and in schools [15]. Interventions delivered digitally are considered to be a key route to scalable and sustainable personalized nutrition interventions [16,17]. Health systems, in particular, play a critical role in the prevention, management, and control of NCDs. The WHO Best Buys Guide [18] recommends that health systems prioritize implementation of nutrition education and counselling to increase fruit and vegetable intake and physical activity. Primary care is especially well positioned to address primary and secondary prevention of NCDs [19]. However, the implementation of multi-factor lifestyle-related intervention in primary care often has limited effectiveness [20,21]. Carrying out primary prevention and health promotion activities such as offering personalized dietary advice is challenging. Effective implementation of personalized dietary advice is influenced by many factors, including professionals’ knowledge, beliefs and experiences; patients’ attitudes; value given by managers and senior leaderships; and availability of longer consultation times and (local) referral resources, among others [22,23].

In the Basque Country, Spain, on-going systematic research has been examining the effectiveness of a lifestyle behavior change intervention and implementation strategies for the sustained use of this intervention in primary care (e.g., [24,25]). This “Prescribing Healthy Life” [“Prescribe Vida Saludable” (PVS) in Spanish] program involves an intervention based on the 5 As intervention framework—Ask, Advise, Agree, Assist, and Arrange follow-up [26,27]—centered on personalized dietary advice, physical activity, and smoking cessation. As part of the intervention, there is an Information and Communication Technology (ICT) tool integrated into the electronic medical record that is used for baseline data collection, tracking and monitoring of individual progress, and to record the personalized prescription(s) or treatment plan for behavior change. PVS also has a community component, led by primary care in local neighborhood health centers, in order to form learning communities to address wider socio-environmental determinants of healthy lifestyle behaviors [28].

This study examines a sub-set of qualitative data collected from focus groups with two different groups: primary health care center professionals and patient participants involved the Phase III quasi-experimental hybrid effectiveness–implementation design trial of the PVS program. The objective of this study was two-fold: (1) to determine barriers and facilitators, *as perceived by health care professionals*, of PVS program implementation specific to the personalized dietary advice component of the intervention and (2) to understand the barriers and facilitators *perceived by PVS program participants* regarding the implementation of the personalized dietary advice component of the intervention in primary care.

## 2. Materials and Methods

*Participants*: Between May 2016 and January 2018, 7 primary health care centers in the Bilbao, Spain metropolitan area implemented the PVS program. In March and April of 2018, professionals from participating PVS centers were recruited through existing PVS program contacts at each center to participate in focus groups to evaluate the program. To accommodate schedules, these focus groups were planned on site at the center and most of them were held over the lunch break so that professionals from both morning and afternoon shifts could participate. Two groups were held at one center to allow all interested professionals to participate. In total, 49 professionals from 6 centers participated in 7 focus groups to evaluate the program, with an average of 7 professionals per group (range 5–11). See breakout of the types of professionals participating in Table 1.

In April, May, and June of 2018, PVS program participants were recruited to participate in focus groups to discuss barriers and facilitators to healthy lifestyles and the role of their local health center in health promotion. As part of a different, but related, study on the effectiveness of the PVS Program, participants were randomly selected to participate in a telephone survey to determine if they had changed any of the three lifestyle behaviors targeted in PVS. For this study, a random sample of individuals who completed the telephone survey were recruited to participate in the focus groups. The focus groups were held in downtown Bilbao or in the town of the health center, depending on distance from Bilbao. They were held after work, so that all those who wanted to participate could join. Six focus groups were held for the PVS program participants of the six centers that carried out the 5 As intervention to discuss barriers and facilitators associated with implementation and behavior change. In total, 47 PVS program participants participated in 6 focus groups, with an average of 8 participants per group (range 7–9). The socio-demographic characteristics of the sample are presented in Table 2.

*Data collection*: Facilitator’s guides were developed for each set of focus groups. The facilitator’s guide for the health center professional focus groups was based on the Consolidated Framework for Implementation Research (CFIR) [29]. The CFIR is used extensively in implementation research because it structures constructs associated successful evidence-based practice implementation into five domains: (1) intervention characteristics, (2) outer setting (e.g., the community and health system), (3) inner setting (e.g., the health center), (4) characteristics of the individuals involved (e.g., the health center professionals), and (5) implementation process. It has been used in prior phases of the PVS research program and was found to be useful to guide future implementation phases [30]. The facilitator’s guide for the PVS participant focus groups was designed to explore: how participants defined healthy lifestyles; their current habits with respect to diet, exercise, and smoking; their attempts to change these behaviors; and the role of primary care in health promotion activities.

This study received ethics committee approval from the Basque Government Ethics Committee (CEIC PI2019117). All participants provided signed informed consent at the beginning of each focus group session agreeing to be recorded and could opt to leave this study at any time. The focus groups lasted 90–120 min, with the groups of professionals lasting longer than the groups of PVS participants. One researcher (SPH) moderated all of the focus groups, with another researcher (HLR) observing them and taking detailed notes. Each focus group was audio-recorded and transcribed verbatim. The two researchers involved in data collection compiled post-session notes.

*Data analysis*: The transcriptions from the professionals were coded line by line by two (SPH and HLR) using ATLAS.ti version 7 (ATLAS.ti Scientific Software Development GmbH, Berlin, Germany), with involvement of a third coder (GG) as necessary for consensus. They applied the CFIR constructs within the five domains using the operational definitions and inclusion/exclusion criteria for each construct available at https://cfirguide.org/constructs/ (accessed on 27 July 2021). Quotations could be assigned more than one construct. Next, the codes were exported into Excel and rated by two coders (HLR and SNF) according to the degree of association with successful implementation from −2 to + 2 (instructions available here: https://cfirguide.org/evaluation-design/qualitative-data/, accessed on 27 July 2021). This valence determined if the construct identified for the quotation was a barrier (negative valence) or facilitator (positive valence). This paper’s aim was to examine the personalized dietary advice component of the PVS intervention. Thus, the same two coders (HLR and SNF) extracted quotations that either (1) directly referred to diet/nutrition and/or using that terms or describing food, eating behaviors, places to eat or buy food, or nutritionists; or (2) directly referred to this topic by mentioning overweight or obesity. Any discrepancies were resolved via consensus.

As part of a larger participant study, the transcriptions from the PVS participants were coded line by line by two coders (HLR and SNF) to identify the type of behavior referred to (either, diet, exercise, or smoking), to determine the appropriate construct(s) according to the Theoretical Domains Framework [31], and to assess if that construct was a barrier or facilitator related to the behavior in question. Any discrepancies were resolved via consensus. As this paper’s aim was to examine eating behaviors, diet, and nutrition, the sub-set of quotations from the transcripts that specifically addressed this topic were extracted for inductive coding [32]. Two researchers (HLR and SNF) carried out initial coding and applied descriptive labels generated by the data, including if the label was a barrier or facilitator. These labels were refined in an iterative fashion and eventually hierarchically organized as parent and child codes. HLR lead the development of the final codebook, which included the code, a definition of the code, and how it contrasted from other codes in the book. Both researchers discussed discrepancies in coding, which supported the refinement of the codes and definitions. Each quotation was coded independently by each researcher, who were blinded to one another’s work until coding was complete. Quotations could be assigned more than one code. Discrepancies in final codes were resolved via consensus. Thematic analysis [33,34] was used to categorize groups of codes into meaningful themes.

## 3. Results

The full results are presented in Table A1 and Table A2 in tables that provide the constructs grouped by domain/theme, the number of barriers and the number of facilitators within that construct/theme, and an example quotation to illustrate the construct/theme. Table A1 describes the barriers and facilitators of implementation of the personalized dietary advice component of a lifestyle intervention in primary care, according to the Consolidated Framework for Implementation Research (CFIR) Domains and Constructs. Table A2 identifies the themes describing the barriers and facilitators as perceived by the PVS program participants. The results section of this paper highlights the most important findings.

### 3.1. Findings from the Professionals Who Carried Out the Personalized Dietary Advice Intervention

**Figure 1:**
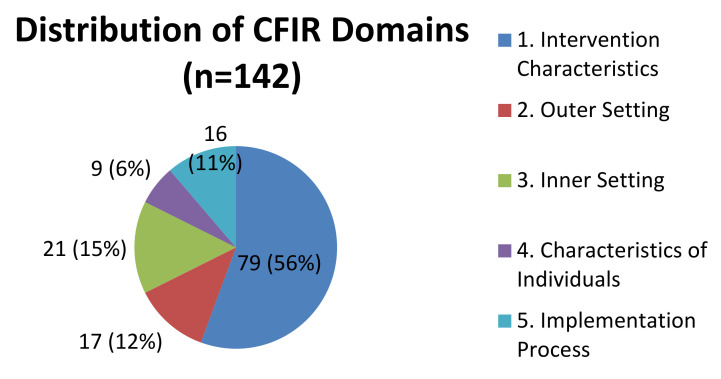
The Relative Importance of the Five CFIR Domains for Professionals.

Over half of the 142 coded quotations addressed constructs in Domain 1. Intervention Characteristics, while the other domains represented 6% to 15% of the quotations each. See Figure 1. The proportion of barriers and facilitators were split fairly equally across most of the CFIR domains, with slightly higher proportions of barriers than facilitators in four of the five domains. In Domain 4. Characteristics of Individuals, 89% of the quotations were facilitators. Results from each of the domains are elaborated in order of importance.

#### 3.1.1. CFIR Domain 1. Intervention Characteristics

Constructs in this domain described the intervention characteristics. Quotations were split between barriers (51%) and facilitators (49%). The barriers related to the design and packaging of the intervention, which was discussed most, and the complexity of the intervention. The facilitators included high evidence strength and quality supporting the intervention, high adaptability and high relative advantage.

Specifically, regarding design and packaging, the participants found the ICT tool was not adequate for tracking small dietary changes. One professional described the barrier in this way:

*“So, for healthy diet, when you re-evaluate after the prescription, the ICT tool only allows the quantities of fruits and vegetables to sum to five. Imagine that you have a patient and you ask him/her “How many fruits and vegetables do you eat?” and he/she responds “None” and you ask “And vegetables?” and he/she says “None”. So you write a prescription and go to evaluate compliance with the treatment. And the patient tells you “I didn’t eat any fruit before, but now I’m eating two fruits a day!” and you know this is an improvement, but you don’t have any way to capture that because when you input two fruits and no vegetables, the patient doesn’t reach the minimum of five pieces and it’s an improvement achieved over 6 months but it shows as though he/she is non-compliant. The ICT tool never shows that you wrote the prescription and that the patient still hasn’t reached five pieces a day, but he/she has increased intake to two pieces of fruit per day when he/she didn’t eat any before.”*

The dietary advice component of the intervention was found to be challenging, especially with respect to the shared decision making required that takes skill and time. Complexity was an important barrier for the professionals in primary care, as illustrated by one professional in this quote:

*“Think about how you get to that result (a prescription for healthy lifestyle behavior). You have a lot of his/her information to go over with the patient and agree on, to ensure that you both agree what that means. It takes time to have a conversation, to learn about what he/she is willing to do, and how he/she is willing to do it. This all takes time. And there’s the patient, who’s a little scared by all this. It seems as if you’re asking him/her to sign a contract, that you promise to (eat) three pieces (of fruit) a day, or one spoonful of olive oil. Sometimes it isn’t easy to do this in one consultation. It takes a lot of time, and that’s what I’m trying to tell you, it’s all that. It usually takes me 20 to 30 minutes just to do the baseline surveys with the patient, so you really need a lot of time to be able to do all the steps.”*

At the same time, the professionals described important facilitators of effective implementation. In particular, it was important to have documented evidence strength and quality, which was evident from prior research by the PVS research team [35,36] and also from direct experience observing the impact of the intervention on their patients. One professional stated:

*“I think it’s fundamental for you to believe in it (the intervention) and see its utility. And then, what (colleague) said, that you can see the results on health too. I think that it’s (the value of the intervention) clear—at least, in most cases, we would avoid high rates of obesity.”*

Furthermore, given the personalized nature of the advice, the intervention was viewed as having sufficient adaptability to be used with patients with a variety of chronic conditions, as well as healthy ones. Another professional commented:

*“But has this (intervention) helped you? It depends on the pathology. With a diabetic patient, you talk about a special diet for diabetes management. This is more general, but it’s also valuable for a diabetic patient. You can go over the (food) pyramid and all that.”*

The personalized dietary advice component of the intervention was evaluated as being superior to other similar programs. Additionally, the ICT component played a critical role in this opinion. One professional enthusiastically endorsed the relative advantage of the intervention, saying:

*“I was really excited by the thought of being able to get better results than I was getting using other methods. (The intervention) It’s similar, but a little more structured (than others). I liked that it had lots of information to help give advice, documents and such. And, on one hand, the (ICT) tool took up my time, but, on the other hand, it facilitated (the interaction). You had all of the possibilities (in the ICT tool)—you could offer diet X, or give advice and it was all well-elaborated. I like all these things about the PVS program.”*

#### 3.1.2. CFIR Domain 3. Inner Setting

Constructs in this domain described the conditions of the health center in which the intervention was being implemented. The quotations were split between barriers (62%) and facilitators (38%). The facilitators included compatibility with the values of primary care professionals, the relative priority of the intervention compared to other programs the center was involved in, and the learning climate in the center in which the strengths and interests of individual professionals were leveraged to successfully carry out the intervention. One participant explained the relative priority of the intervention and his/her patient’s response in this way:

*“We were obsessive about it (carrying out the intervention). My patients asked if I owned a fruit stand in town or something because I told one women that (her intake of) vegetables were very good, but she needed to eat a little more fruit. And she asked me if I had a fruit stand in town. And I said no ma’am, I just see that you could use a little more fruit in your diet. You have to start the conversation this way with some people, you know.”*

The barriers centered on networks and communication, where newly incorporated professions did not hear about the intervention, and implementation climate, where the whole team of professionals was not on board in carrying out the intervention. Additional important barriers were related to available resources such as time and training. The personalized dietary advice component of the intervention was viewed as not compatible with existing workflows in primary care, as illustrated by this comment:

*“There are parts (of the intervention) that you can take on. Smoking cessation you can take on (but not personalized dietary advice or exercise) with the schedules that we have, with the visit times that we have.”*

#### 3.1.3. CFIR Domain 4. Outer Setting

Constructs in this domain described the conditions outside the health center, such as the health system or the community, in which the intervention was being implemented. Quotations were split between barriers (65%) and facilitators (35%). Facilitators included cosmopolitanism, which related to existing relationships with the community regarding offering dietary advice. Barriers related to external policy and incentives, where the health system did not adequately support providing substitute professionals to cover missed time or the extra time spent dedicated to carrying out the intervention. Other barriers were identified with respect to the extreme needs and (lack of) resources regarding dietary advice, as illustrated in this explanation:

*“I was really surprised. There are kids who don’t eat fruit or vegetables. And there’s no way to make them eat them, either. A kid comes in who doesn’t eat practically any fruits or vegetables because he/she doesn’t like them. At that point, there is no way you’re going to get that kid to eat (fruit or vegetables). For the kids that do eat three portions, it’s easy to encourage them to eat more, but the kid who doesn’t eat fruit because he/she doesn’t like it isn’t going to start eating it. Just like an adult might be—it’s like “mission impossible”. It’s also “mission impossible” to get kids to eat five portions (of fruit and vegetables) when they eat in the school cafeteria. At night, eating so much fruit and vegetable isn’t reasonable for the kids who eat in the cafeteria and the parents have less influence over that meal.”*

#### 3.1.4. CFIR Domain 5. Process

Constructs in this domain described the implementation process. Quotations were split between barriers (63%) and facilitators (37%). Important facilitators were identified with respect to engaging key stakeholders (the professionals themselves) by allowing different professionals to deliver different aspects of the intervention depending on their interest areas and availability. Barriers related to appropriate planning of intervention delivery and difficulty engaging the innovation participants (health system users). Various barriers engaging external change agents were also described. For example:

*“They (community agents) were involved. Even the restaurants went to the schools with healthy snacks. This (intervention) isn’t just in the health center—it is in the health center, the schools, the pharmacies. And so then it is a little more powerful. You act, and the others support. It’s true that there are some things that don’t depend on us. If there’s a vending machine at the school and kids can buy sugary snacks like doughnuts or whatever, then it’s difficult. But if we’re all going in the same direction, it’s easier. What’s bad about this is that it has to involve the school, and a lot of people (community agents) have to be involved, because if you are able to do something in one place, but it’s the opposite everywhere else, it doesn’t work.”*

#### 3.1.5. CFIR Domain 4. Characteristics of the Individuals Involved

Constructs in this domain described the characteristics of the health center professionals carrying out the intervention. Quotations were almost all facilitators (89% vs. 11% barriers). The individual stage of change of the professional, which is related to self-efficacy, was a key facilitator. Other facilitators were related to knowledge and beliefs about the intervention and its expected impact, as one professional describes below:

*“For us, as health center personnel, I think it (PVS) has been beneficial to us personally. At first, when we told our patients to eat five servings of fruits and vegetables, (we thought) no one would eat that, and do so much exercise. Uffff. And now it’s on our minds at home too. At home my family tells me that I’m crazy. I’ve internalized (the recommendations) to the point that if we don’t eat five, we eat four (rations). It has benefited me.”*

### 3.2. Findings from the PVS Participants Regarding the Personalized Dietary Advice Intervention

The most important themes that emerged from the discussion groups with the PVS program participants related to (1) the importance of personalization of advice (both barriers and facilitators), (2) the value of follow-up with feedback (majority facilitators), (3) seeing the outcomes achieved as a result of personalized dietary advice intervention (majority facilitators), and (4) the preparedness and role of primary care to provide personalized dietary advice (majority barriers). These major themes are described in detail below. Full results can be found in Table A2.

#### 3.2.1. Importance of Personalized Advice

One-fourth of the coded quotations (25%) fell into this major theme in which general advice was seen to facilitate improvement in healthy eating behavior (62% facilitators vs. 38% barriers), while a lack of personalization was identified as a barrier (61% facilitators vs. 39% facilitators). Participants wanted personalized advice in terms of what to eat and how to incorporate healthy eating into their daily activities. For instance, one participant explained:

*“I just didn’t want to go any more (to the health center for advice) or try to help myself (lose weight) because they didn’t show me anything. So I said I won’t go. Then, the other day, I went to have bloodwork done and was in there 5 min. They took my blood pressure and told me to come back next month…but then, later on, I spent 20 minutes with the nurse. She started to explain that you need to eat this and that…she talked for half an hour about what I should be eating and I didn’t eat any of that. In fact, I wanted to throw up.*

*They print off a ton of papers. And they’re very strict about what to eat. An apple with a small piece of bread and coffee. And I said, and what if I don’t want to eat breakfast or I don’t like that food? Why do I have eat that for breakfast? They want to help, but have little idea how (to help).”*

#### 3.2.2. Value of Follow-Up

Approximately one-fifth of the coded quotations (18%) fell into this theme in which participants recognized the importance of regular follow-up and feedback to continue following the personalized dietary advice received (69% facilitators vs. 31% barriers). The obligation associated with having a follow-up appointment was seen as a driver of compliance with the dietary advice received. One participant described this in the following way:

*“Follow-up is important, especially continuous follow-up at the beginning, because you go and want to lose weight. If, the next month, you’re back in to see her and you are losing the weight and she gives you other steps and tells you what you need to do and you see that you’re doing well. (It’s different) if they tell you, we’ll see you in six months. Then you forget about it right away or you get bored. But, if, at the beginning, you start to catch on, it’s easier and you keep doing it.”*

#### 3.2.3. Seeing Outcomes Achieved

Fifteen percent of the coded quotations fell into this theme, which included achieving desired outcomes such as losing weight or changing eating behavior and being satisfied with care (75% facilitators vs. 25% barriers). Participants stated how happy they were with their primary care physician (PCP) because of the quality of care, how they were able to make radical lifestyle changes, and how surprised they were that they were able to lose considerable weight in the first month of the intervention. See example quotations of these themes in Table A2.

#### 3.2.4. Perceptions Concerning the Preparedness and Role of Primary Care

Twelve percent of the coded quotations fell into this theme, in which the participants perceived that primary care was the place to receive personalized dietary advice, but that it was not being carried out effectively (79% barriers vs. 21% facilitators). Similar to the findings from the professionals, PVS participants felt that health promotion and disease prevention was part of the role of primary care, but that primary care lacked preparedness to offer personalized dietary advice. In particular, participants noted that professionals lack sufficient time to spend with them, had high workloads, and lacked adequate training. One participant said:

*“The foods that you can eat, and those that you can’t, they (primary care professionals) tell you a little bit of everything. It would be interesting too, just like they do with the mammogram and those things, to do this (healthy lifestyles) too. An expert—not a physician, because (physicians) are there for other things, like to diagnosis illnesses, and they have too much work sometimes. Sometimes I go, and I’m in and out in 5 min, but other times it takes half an hour. Of course it’s complicated for them (primary care professionals) to dedicate half an hour to each and every patient. And finding the time to tell a patient what he/she should eat, what he/she should do. Of course it’s difficult for them (primary care professionals).”*

The minor themes that emerged from the participants included more prevention (including screening and early intervention) efforts, less punishment and more reinforcement (including a desire to pay for this service offered free by the universal health care system in the Basque Country, Spain), general support and encouragement, more multi-disciplinary and psychological support, and a guarantee that all professionals are on the same page with respect to the advice given. Trust (including evidence of effectiveness), which can stem from the long-term PCP–patient relationship, was identified as a facilitator of effective dietary advice implementation. See example quotations of these themes in Table A2.

## 4. Discussion

Both professionals and participants described perceived barriers and facilitators to the implementation of the personalized dietary advice component of a lifestyle change intervention carried out in primary care. Professionals identified slightly higher proportions of barriers than facilitators across four of the five CFIR domains. The intervention characteristics domain was most important, emphasizing barriers in the design and packaging and complexity, while citing evidence strength and quality, adaptability, and relative advantage as facilitators. PVS program participants felt that personalized advice was important, but that more personalization was needed from the professionals in primary care. Participants received follow-up with feedback and noted that this, along with seeing the results, facilitated adherence to the dietary advice. Both professionals and patients described primary care as the appropriate place for personalized dietary advice, but that lack of time, workload, and sometimes training were barriers to effective implementation.

Regarding intervention characteristics, previous research with health professionals implementing lifestyle behavior change interventions supports complexity as a barrier because of the many steps or components of the intervention, from initial assessment, to the patient being willing to change, to agreeing on a personalized treatment plan (e.g., [37]). Patients agreed, but expressed the need for even more personalized advice that specifically addressed how to incorporate new eating behaviors into their daily routines. The ICT tool, within the design and packaging aspect of the intervention, was described as valuable for tracking and monitoring progress, similar to those reported by Berman et al. [16]. However, professionals carrying out the personalized dietary advice component of PVS wanted the tool to be more sensitive to capturing small changes in participants’ diets. They found the tool and intervention helpful to tailor prescriptions to the patient’s needs, making the intervention adaptable to patients with different chronic conditions. Relative advantage of the program was found to be a facilitator in this study, and was also important to successful implementation of a weight loss management program (MOVE!) [38]. Evidence strength and quality was viewed by professionals of this study as an important facilitator in this study. In contrast, Alagell et al. [37] found that PCPs questioned the evidence concerning the effectiveness of multiple health behavior change interventions and were skeptical of the success of such programs implemented in primary care settings. Prior research by the PVS research team in the Basque Country documenting intervention effectiveness may explain these differences in findings [24,25,28,30,35,36].

Evidence strength and quality is closely related to knowledge and beliefs, a key characteristic of the individual found to facilitate implementation of the personalized dietary advice component of the intervention. Previous research highlights the importance of this factor [37]. Brotons et al. [39] interviewed PCPs in 11 European countries and found that those who smoked felt less able to provide effective smoking cessation counselling and those who were sedentary believed they were less effective in offering physical activity advice. In contrast to the findings of the present study, the results of a scoping study [40] including PCPs in Scotland [41] specifically indicated that preventative dietary advice was not effective and implementation could damage the physician–patient relationship. Rubio-Valera et al. [22] also support the idea that professionals’ knowledge and beliefs about the intervention is an important factor in implementation. In the PVS program, knowledge and beliefs facilitated successful implementation. This finding may be attributed to the systematic research program being carried out in the Basque Country which has demonstrated the effectiveness of the PVS intervention in prior randomized controlled trials [24,42,43,44] and the effectiveness of the implementation strategies engaging key stakeholders in a collaborative fashion [28,35].

Factors related to the inner setting, or health center in this study, are particularly important for successful implementation of health promotion interventions in primary care. In the present study, the relative priority given to the intervention by professionals was a facilitator, such as was found in the study of MOVE! Program implementation [38]. Another important facilitator was the compatibility with values of primary care, as perceived by both professionals and program participants, which coincides with some prior research [22,38,39,45], yet contradicts the scoping review and study of PCPs in Scotland [40,41]. These differences may be explained by the characteristics of the individuals, such as age and gender. In the Scottish study, Fuller et al. [41] reported that female PCPs of younger age believed lifestyle counselling was a core part of primary care and could leverage their credibility and established physician–patient relationship. PVS program participants agreed that a good, personal PCP–patient relationship helped motivate them to change their behavior and created a sense of obligation to carry through with the treatment plan, as found in prior research [46]. Similarly, as Mazza et al. [47] identified, participants indicated that trust, rapport, and continuity of care with their PCP facilitated adherence to the personalized dietary advice component of the PVS program intervention.

The inner setting domain also captured some important barriers to the implementation of personalized dietary advice in primary care. In this study, both professionals and participants engaged in the PVS program perceived a lack of compatibility with workflows and a lack of available resources, specifically time for the visit consultation. Research supports the role of this barrier [22,23,37,39,41,45], and also highlights a heavy workload [39] and lack of referral resources in primary care [22,37]. PVS program participants valued regular follow-up and feedback throughout the intervention, also identified by Celis-Morales et al. [48], and viewed this as helping them to adhere to the personalized dietary advice. At the same time, the participants recognized the lack of professionals’ time available for the longer and/or more frequent consultations required by the intervention, as found by Moreno-Peral et al. [49].

Regarding the outer setting, this study identified a number of barriers regarding the community and health system in which the intervention is implemented. In particular professionals noted the role of needs and resources of the population because of poor dietary intake of fruits and vegetables (especially in children), and external policy and incentives such as the lack of adequate substitutions to cover longer visits or intervention-related training. In contrast, Elwell et al. [23] found that PCPs felt that their patients did not need advice and knew what they needed to eat, whereas, similar to the findings of the present study, patients actually wanted more prevention and early intervention in primary care. Damschroder and Lowery [38] found the MOVE! Weight management program fit patient needs. More research is needed into specific population needs at the level of local neighborhoods, as well as health system policies regarding health promotion and personalized dietary advice, and how these factors might influence successful implementation of personalized dietary advice in primary care. Regarding implementation process, since the PVS program had a strong community component, a key barrier was engaging external change agents over the long-term implementation of the program. Although there is consensus in the literature about the value of multi-sectorial approaches to change population-level health behaviors [2,15], further research into the value of primary care/family and community medicine practitioners as leaders in such a public health effort is warranted.

One strength of the present study is that it captures the perspectives of both professionals and participants involved in implementation of the dietary advice component of a lifestyle intervention program carried out in primary care. This dual perspective offers complimentary, and sometimes contradictory, views. However, the study has an important limitation. The data analysis conducted for this paper represents a shift from the originally proposed aim of the larger study in order to focus on the dietary advice component specifically. For the professionals, it is possible that important barriers and facilitators, for instance with respect to the inner setting, may not have been adequately captured because they were made in more general statements that did not meet the inclusion criteria of being specific to the dietary advice component of the intervention. For the PVS participants, the focus group facilitator guide had a multi-purpose designed, so it is possible that some themes relevant to the topic of the analysis in this paper may not have emerged.

## 5. Conclusions

In summary, both primary care professionals and participants of a lifestyle intervention program endorsed primary care as an appropriate place for personalized dietary advice intervention. However, the dietary advice component of the PVS program involved many steps and was complex for primary care professionals to carry out. Offering dietary advice required time and skill to understand the patient’s baseline dietary intake, agree on the need to change, determine the extent of the patient’s willingness to adopt new eating behaviors, write a personalized prescription, and follow-up accordingly. Although the ICT tool assisted with tracking, monitoring, and prescribing individualized healthy diets, more granular data capture was required. PVS participants wanted the advice to be even more personalized, especially with respect to how they could incorporate recommended changes to their diet into their day-to-day lifestyle. The needs of the population regarding healthy diet were assessed as high by the professionals, and it was difficult to involve external change agents from the community for a successful multi-sectorial approach to healthy diet. Lastly, both professionals and PVS program participants cited the lack of time for visits, heavy workload for the primary care professionals, and sometimes a lack of training, as additional barriers to effective implementation of the personalized dietary advice component of a lifestyle intervention in primary care.

At the same time, there were several facilitators related to successful implementation of the personalized dietary advice component of the PVS program. The availability of high-quality evidence of effectiveness, the adaptability of the intervention as beneficial for healthy individuals and patients with a variety of chronic conditions, and the relative advantage of the PVS program that included the ICT support tool were all positively evaluated by primary care professionals. PVS participants valued regular follow-up and feedback, which they felt helped them comply with the advice received. Knowledge and beliefs about the personalized dietary advice component specifically and its effectiveness, facilitated successful implementation.

The barriers and facilitators identified in the current study are primarily modifiable factors. Implementation strategies to enhance the facilitators and overcome the barriers can be identified and developed in further research. These strategies can be integrated into future personalized dietary advice and personalized nutrition interventions to improve their sustainable use by professionals, increase participant adherence, and achieve a potentially greater impact on population health.

## Figures and Tables

**Table 1:** Type of professionals involved PVS professional sample (N = 49).

Type of Professional Involved in the Study	n (%)
Primary Care Physicians and Pediatricians	24 (49.0%)
Primary Care Nurses and Midwives	18 (36.7%)
Administrative Assistants	7 (14.3%)
Total	49 (100%)

**Table 2:** Socio-demographic characteristics of the PVS patient sample (N = 47).

Characteristic	n (% or SD)
**Sex**	
Male	28 (59.6%)
Female	19 (40.4%)
**Age**	
Mean (SD, Range)	50.1 (11.4, 20–75)
**Education**	
Secondary school studies or degree	10 (23.8%)
Professional studies	20 (47.6%)
University studies or higher	12 (28.6%)

## Data Availability

Data is contained within the supplementary material. Additional data related to the constructs and themes described in the article and supplementary material are available on request from the corresponding author.

## Appendix A

**Table A1:** Barriers and facilitators of implementation of the personalized dietary advice component of a lifestyle intervention in primary care, according to the Consolidated Framework for Implementation Research (CFIR) Domains and Constructs.

Construct	Barrier	Facilitator	Quotation
**1. Innovation Characteristics**	**40 (51%)**	**39 (49%)**	
Evidence Strength and Quality	2 (18%)	9 (82%)	“I think it’s fundamental for you to believe in it (the intervention) and see its utility. And then, what (colleague) said, that you can see the results on health too. I think that (the value of the intervention) is clear—at least, in most cases, we would avoid high rates of obesity.”
Relative Advantage	1 (17%)	5 (83%)	“I was really excited by the thought of being able to get better results than I was getting using other methods. (The intervention) It’s similar, but a little more structured (than others). I liked that it had lots of information to help give advice, documents and such. And, on one hand, the (ICT) tool took up my time, but, on the other hand, it facilitated (the interaction). You had all of the possibilities (in the ICT tool)—you could offer diet X, or give advice and it was all well-elaborated. I like all these things about the PVS program.”
Adaptability	1 (11%)	8 (89%)	“But has this (intervention) helped you? It depends on the pathology. With a diabetic patient, you talk about a special diet for diabetes management. This is more general, but it’s also valuable for a diabetic patient. You can go over the (food) pyramid and all that.”
Complexity	13 (81%)	3 (19%)	“Think about how you get to that result (a prescription for healthy lifestyle behavior). You have a lot of his/her information to go over with the patient and agree on, ensure that you both agree what that means. It takes time to have a conversation, to learn about what he/she is willing to do, and how he/she is willing to do it. This all takes time. And there’s the patient, who’s a little scared by all this. It seems as if you’re asking him/her to sign a contract, that you promise to (eat) three pieces (of fruit) a day, or one spoonful of olive oil. Sometimes it isn’t easy to do this in one consultation. It takes a lot of time, and that’s what I’m trying to tell you, it’s all that. It usually takes me 20 to 30 minutes just to do the baseline surveys with the patient, so you really need a lot of time to be able to do all the steps.”
Design Quality and Packaging	23 (62%)	14 (38%)	BARRIER: “So, for healthy diet, when you re-evaluate after the prescription, the ICT tool only allows the quantities of fruits and vegetables to sum to five. Imagine that you have a patient and you ask him/her “How many fruits and vegetables do you eat?” and he/she responds “None” and you ask “And vegetables?” and he/she says “None”. So you write a prescription and go to evaluate compliance with the treatment. And the patient tells you “I didn’t eat any fruit before, but now I’m eating two fruits a day!” and you know this is an improvement, but you don’t have any way to capture that because when you input two fruits and no vegetables, the patient doesn’t reach the minimum of five pieces and it’s an improvement achieved over six months but it shows as though he/she is non-compliant. The ICT tool never shows that you wrote the prescription and that the patient still hasn’t reached five pieces a day but he/she has increased intake to two pieces of fruit per day when he/she didn’t eat any before.”
			FACILITATOR: “I really like the ICT tool a lot. It is very powerful. It has a lot of content, and that makes it hard to use at first. But I’ve been able to write various prescriptions with it, and I’m just one nurse.”
**2. Outer Setting**	**11 (65%)**	**6 (35%)**	
Needs and Resources	8 (67%)	4 (33%)	“I was really surprised. There are kids that don’t eat fruit or vegetables. And there’s no way to make them eat them, either. A kid comes in who doesn’t eat practically any fruits or vegetables because he/she doesn’t like them. At that point, there is no way you’re going to get that kid to eat (fruit or vegetables). For the kids that do eat three portions, it’s easy to encourage them to eat more, but the kid who doesn’t eat fruit because he/she doesn’t like it isn’t going to start eating it. Just like an adult might be—it’s like “mission impossible”. It’s also “mission impossible” to get kids to eat five portions (of fruit and vegetables) when they eat in the school cafeteria. At night, eating so much fruit and vegetable isn’t reasonable for the kids who eat in the cafeteria and the parents have less influence over that meal.”
Cosmopolitanism	0 (0%)	1 (100%)	“We tried to give talks to the community. The first one was at a school and it went well, but the second one, very few people came. The idea was to give a talk about healthy eating to all the Parent-Teacher Associations at all the schools in (community). So we tried working with the community, but the demand has to come from the group itself. If they ask for it, and find a place for it, these talks would be powerful (tools). But if they don’t (ask for it), it won’t work.”
Peer Pressure	1 (50%)	1 (50%)	BARRIER: “Exactly how they explained it (the intervention) to us. They used (other community) as an example. They talked a lot about how those in that community and the so-and-so association were involved, and restaurants, and bars.” FACILITATOR: “There’s a lot of coordination possible in places in the community outside of the health center. All the community agents would go to a meeting, right? Representatives from the sports center, the pharmacies, they can collect the surveys (to obtain the baseline reading on healthy lifestyle behaviors). The schools would also be involved, for instance, trying to change the meals in the cafeterias.”
External Policy and Incentives	2 (100%)	0 (0%)	“The least they (health system managers) can do is offer substitutions for the professionals. It is the least they can do. Because when you have to see three patients, and cover patients from two other colleagues, you don’t really want to ask the kid if he/she eats fruit or chickpeas… I spent practically all of last summer here (at work), and Spring Break, the Christmas holidays, long weekends, the possibility of getting someone to fill in for you was nil. But staffing is the minimum resource required.”
**DOMAIN 3. ** **Inner Setting**	**13 (62%)**	**8 (38%)**	
Structural Characteristics	1 (50%)	1 (50%)	FACILITATOR: “Since we’re a small town, there’s just one waiting room (in the health center). The (towns)people are very close to the health center. I think that everyone knows about the program (PVS), about exercise, about healthy diet, that (colleague) is there, even if she’s not your nurse, she’ll help you with smoking cession and all that. For good or bad, (the size of) the town is reflected in the design of the health center with just one waiting room.”
Networks and Communication	1 (100%)	0 (0%)	“I was the last (of the personnel) to arrive (at the health center). I’ve been here one year and no one told me anything about PVS. When the patient comes with the completed survey, I’m supposed to fill in the four key data points (in the ICT tool)—if they smoke or not, if they eat vegetables and/or fruit, and the exercise they do.”
Implementation Climate	2 (100%)	0 (0%)	“At the beginning, it seemed that people were starting to get motivated and it (the program) was beginning to work. A leader was named and we started the project. But the project was like all projects. At the beginning, the whole team wasn’t on board, it is only part of the team (participating). And it wasn’t until we started functioning with the ICT tool, we started meeting (as a group with the external support team) and talking about healthy diet, exercise, smoking cessation, etc. that the project really got off the ground. Various months went by until we were (doing the project with) the society, with the external agents in the community, etc.”
Implementation Climate 2A. Compatibility—Values	0 (0%)	4 (100%)	“When they originally pitched the PVS project to us, it seemed wonderful. It focused on smoking cessation, health eating, exercise, and got everyone working (on this). It seems essential, amazing.”
Implementation Climate 2B. Compatibility—Workflow	7 (88%)	1 (12%)	“There are parts (of the intervention) that you can take on. Smoking cessation you can take on (but not personalized dietary advice or exercise) with the schedules that we have, with the visit times that we have.”
Implementation Climate 3. Relative Priority	0 (0%)	1 (100%)	“The facilitator says: What priority have you given PVS in your center?
			Sure, we’ve given it priority. A lot!
			The facilitator says: And with respect to other initiatives in your center?
			We were obsessive about it. My patients asked if I owned a fruit stand in town or something because I told one women that (her intake of) vegetables were very good, but she needed to eat a little more fruit. And she asked me if I had a fruit stand in town. And I said no ma’am, I just see that you could use a little more fruit in your diet. You have to start the conversation this way with some people, you know.
Implementation Climate 6. Learning Climate	0 (0%)	1 (100%)	“We’re each motivated by something, someone is more into smoking cessation (for example). So, as you go, you see if you can leverage this interest. (One colleague) said “How can we reach the community? We can send more surveys, do that and the other. (Another colleague) can focus on nutrition, figure out how to do it, prepare a talk, go out to the schools, describe healthy diet. So it’s the team (carrying out the intervention) but also the people.”
Readiness for Implementation 2. Available Resources	2 (100%)	0 (0%)	“It would be good to have training in nutrition and exercise, because sometimes the patients ask for more information, about running for instance, and I don’t know how to respond.”
**4. Characteristics of Individuals**	**1 (11%)**	**8 (89%)**	
Knowledge and Beliefs	0 (0%)	4 (100%)	“For us, as health center personnel, I think it (PVS) has been beneficial to us personally. At first, when we told our patients to eat five servings of fruits and vegetables, (we thought) no one would eat that, and do so much exercise. Ufffffff. And now it’s on our minds at home too. At home my family tells me that I’m crazy. I’ve internalized (the recommendations) to the point that if we don’t eat five, we eat four. It has benefited me.”
Self-Efficacy	1 (50%)	1 (50%)	FACILITATOR: “For those that already eat three servings (of fruit), it’s easy to encourage them to eat more.”
Individual Stage of Change	0 (0%)	3 (100%)	“It’s a way to recommend healthy diet and exercise and based on something tangible that has been demonstrated (to work). And that’s why I liked it and it motivated me (to implement it).”
**5. Process**	**10 (63%)**	**6 (37%)**	
Planning	1 (100%)	0 (0%)	“When we started to meet with the community, and we did this outside of the health center, and we all started talking about the same thing, healthy diet, how important a healthy diet was, the mortality associated with certain lifestyle habits, etc. Then people started to get bored. The projects didn’t materialize. Everyone spoke in general terms and the external (community) agents stopped coming. The ones from school said that they weren’t going to come anymore, that they have things to do, and that they didn’t see any progress. So, at the end of the day, like (colleague) said, we were the only ones left.”
Engaging	1 (50%)	1 (50%)	BARRIER: “It doesn’t matter to me if it’s a nutritionist or nurse, but it should be someone who can orient them (towards behavior change) in this area, not just give information about healthy diets, but help them (change).
			The facilitator says: “So you would want to have one person solely dedicated to the prescription and follow-up steps (of the intervention?)”
			To do the follow-up more than other activities
			The facilitator says: “So, for a program like PVS to be successful, a professional would be necessary?”
			Patients come and tell you, “I don’t like bananas” and then you don’t know what to do… a (trained) professional would know how to handle those situations.”
Engaging 4. External Change Agents	2 (67%)	1 (33%)	“At the community level, we tried to give talks to the community. The first one was at a school and it went well, but the second one, very few people came. The idea was to give a talk about healthy eating to all the Parent-Teacher Associations at all the schools in (community). So we tried working with the community, but the demand has to come from the group itself. If they ask for it, and find a place for it, these talks about be powerful (tools). But if they don’t ask for it, it won’t work.”
Engaging 4. External Change Agents 4.1. Community	4 (67%)	2 (33%)	“They (community agents) were involved. Even the restaurants went to the schools with healthy snacks. This (invention) isn’t just in the health center, it is in the health center, the schools, the pharmacies. And so then it is a little more powerful. You act, and the others support. It’s true that there are some things that don’t depend on us. If there’s a vending machine at the school and kids can buy sugary snacks like doughnuts or whatever, then it’s difficult. But if we’re all going in the same direction, it’s easier. What’s bad about this is that it has to involve the school, and a lot of people (community agents) have to be involved, because if you are able to do something in one place, but it’s the opposite everywhere else, it doesn’t work.”
Engaging 5. Key Stakeholders	0 (0%)	1 (100%)	“We’re each motivated by something, someone is more into smoking cessation (for example). So, as you go, you see if you can leverage this interest. (One colleague) said “How can we reach the community? We can send more surveys, do that and the other. (Another colleague) can focus on nutrition, figure out how to do it, prepare a talk, go out to the schools, describe healthy diet. So it’s the team (carrying out the intervention) but also the people.”
Engaging 6. Innovation Participants	1 (100%)	0 (0%)	“Patients who were interested in the topic of diet/nutrition were kinda stuck (because) there wasn’t any feedback.”

**Table A2:** Barriers and facilitators of implementation of the personalized nutrition component of a lifestyle intervention in primary care as perceived by program participants.

Code	Barrier	Facilitator	Quotation
**THEME: Trust**	**2 (22%)**	**7 (78%)**	
Evidence of effectiveness	1 (33%)	2 (67%)	“I have friends who are doing diet X and they’ve lost weight in three months. And of course you’ll see an effect—I don’t have a clue why they do the artichoke one. I don’t doubt why they’ve lost weight, but long term? I’d like a long-term diet to maintain my weight at X kg. I don’t want to lose 10 kg quickly, just to add 50 kg back on a year or two later. That’s crazy! That’s why I prefer a physician. I trust him more.”
Trust	1 (17%)	5 (83%)	“Regarding changing bad habits, I stopped smoking. You know, it was the cigarette after coffee that got to me. And I got stressed out. So I spoke with my PCP, because all this is with the physician. You can’t do whatever you want. I have anemia, so I can’t stop eating certain foods, although I’d like to, because I don’t like them, but I have to eat them. So I changed my eating habits too. I stopped drinking coffee and started drinking tea. I started to eat fruit. It all depends on the amount of work you have. If you’re tired, you won’t eat an apple. Talking about five meals a day, that has always stressed me out. I’ve never done it. But in the morning I started eating fruit, five real fruits. When you have problems you can’t do whatever you’d like…”
**THEME: Same Page**	**3 (60%)**	**2 (40%)**	
Same page	3 (60%)	2 (40%)	“My PCP told me that I had to each five pieces of fruit a day. And I ate five pieces. I stopped eating junk food and all that. Then I went to have more blood work done and my PCP wasn’t there. But he left a note asking me if I stopped eating this or that and how many pieces of fruit I ate. I said “Five” and the substitute PCP said “Five? That’s a lot!” I wish they’d get on the same page. Because one (PCP) tells me one thing and another (PCP) tells me another. I don’t know if they don’t explain it right, or we understand it wrong—probably a little of both!”
**THEME: Additional Needs from Primary Care**	**8 (57%)**	**6 (43%)**	
Crack the whip	0 (0%)	1 (100%)	“The other day I had some blood work done and he (my PCP) started cracking the whip. He got on me a few years ago (about eating better). And now I’m on the right path, losing (weight) little by little.”
Encouragement	3 (50%)	3 (50%)	BARRIER: “I’d like some more attention, encouragement (from my PCP), asking me what I’m eating…I don’t know, a chat (about this), at least, but a little chat would help a lot, I think. It’s not his fault. Later it’s your decision, but then you get discouraged.”
			FACILITATOR: “I thought I was going to weigh in at 116 (kg) because I was at 114 (kg) when I went last time. But she encouraged me. She said “Good! You haven’t gained weight! And you’ve stopped smoking and it’s normal.” She encouraged me to keep going. Once I went from 115 (kg) to 107 (kg) and she said that the scale must be wrong. She got on it, and it was correct. She really did encourage me, and I liked it. I don’t have any complaints.”
Support	5 (71%)	2 (29%)	“For instance, if I want to lose weight, (my PCP) could give me some tips, help me along, send me for bloodwork, recommend a dietician to learn to eat better. (I’d like) some support, in this case for this reason (to lose weight). If you have cancer, you don’t have a physician, you get diagnosed and you see an oncologist and you have treatment. It seems like, for healthy lifestyles, which underlies everything, we know the theory but not the practice. It’s complicated, but it might make everything much easier. We wouldn’t need medications. Why take pills if you can fix the issue and not gain as much (weight)?
**THEME: Prevention**	**13 (72%)**	**5 (28%)**	
Screening	2 (67%)	1 (33%)	“The pediatrician should ask what you eat, how much sugar you eat. That’s the problem. You have to teach them when they’re little… And this is critical, and pediatricians can really do something. Ask what they eat, what they snack on, what they have for breakfast, that’s really important. There are kids who go to school without eating breakfast. They take a sandwich, or junk food (with them for snack).”
Early intervention	3 (75%)	1 (25%)	“But eating only salads? Surely that’s not good. Sometime I eat them at night, but always with something else, I suppose. It’s the lack of information, beginning with schools. For instance, (the health system) or the government ought to give talks to kids about what they should eat. We should learn, and I think it should all start (when they are young), like everything else.”
Prevention	8 (73%)	3 (27%)	“I’ll tell you one thing, until something’s happened to you (medically), you don’t find out that that (food) pyramid exists. That’s what I’m talking about. Now you have to eat this or that because look what’s happened to you. It’s what’s happened to all of us. You’ve been eating a certain way for 50 or 60 years, and now you have to correct what you’ve been doing your whole life and (make the change) in 5 or 10 years. There you have it. We get the information after the scare. When you have your bloodwork results back, look, you have to eat this, and this, and this.”
**THEME: Advice**	**31 (53%)**	**28 (47%)**	
Advice	8 (38%)	13 (62%)	“Then I went to the doctor and he started to weigh me and take my blood pressure. Then, what I started to do is to weigh what I ate that I thought could make me gain weight. And, of course, when I get home from work, it’s easy to grab a sandwich. And the doctor told me, it’s a small sandwich, but, at the end of the day, it’s still a sandwich. So then I began to change, (eating) soy-based yogurt instead of milk-based, and (I) stopped eating sandwiches and ate fruit.”
Personalized	23 (61%)	15 (39%)	“I just didn’t want to go any more (to the health center for advice) or try to help myself (lose weight) because they didn’t show me anything. So I said I won’t go. Then, the other day, I went to have bloodwork done and was in there 5 min. They took my blood pressure and told me to come back next month…but then later on, I spent 20 minutes with the nurse. She started to explain that you need to eat this and that…she talks for half an hour about what I should be eating and I didn’t eat any of that. In fact, I wanted to throw up.
			They print off a ton of papers. And they’re very strict about what to eat. An apple with a small piece of bread and coffee. And I said, and what if I don’t want to eat breakfast or I don’t like that food? Why do I have eat that for breakfast? They want to help, but have little idea how (to help).”
**THEME: Follow-up**	**13 (31%)**	**29 (69%)**	
Follow-up	4 (25%)	12 (75%)	“And the follow-up with the physician or specialists at the health center, they follow-up with you and you feel an obligation towards them to go to the follow-up visit and follow their advice, and basically that’s it.”
Feedback	9 (35%)	17 (65%)	“Follow-up is important, especially continuous follow-up at the beginning, because you go and want to lose weight. If, the next month, you’re back in to see her and you are losing the weight and she gives you other steps and tells you what you need to do and you see that you’re doing well. (It’s different) if they tell you, we’ll see you in six months. Then you forget about it right away or you get bored. But, if, at the beginning, you start to catch on, it’s easier and you keep doing it.”
**CODE**	**BARRIER**	**FACILITATOR**	**QUOTATION**
**THEME: Perceptions of Primary Care**	**23 (79%)**	**6 (21%)**	
Perceived preparedness of primary care	8 (80%)	2 (20%)	“The foods that you can eat, those that you can’t, they (primary care professionals) tell you a little bit of everything. It would be interesting too, just like they do with the mammogram and those things, to do this (healthy lifestyles) too. An expert—not a physician, because (physicians) are there for other things, to diagnosis illnesses, and they have too much work sometimes. Sometimes I go, and I’m in and out in 5 min, but other times it takes half an hour. Of course it’s complicated for them (primary care professionals) to dedicate half an hour to each and every patient. And finding the time to tell a patient what he/she should eat, what he/she should do. Of course it’s difficult for them (primary care professionals).”
Perceived role of primary care	15 (79%)	4 (21%)	“Along the lines of what the others are saying about telling you a little about the foods that you can and can’t mix, sometimes there are people who are intolerant (to certain foods) and we don’t know. We’re trying to eat right, but we’re intolerant and doing it wrong. It’s not easy…. But if we’re intolerant to a certain food, it would be interesting (to know), because most people don’t have any idea that this food or that food doesn’t sit well with me. And it turns out I’m lactose intolerant and I didn’t have a clue. We shouldn’t have to wait 50 or 60 years to find out that my body can’t handle milk, or certain kinds of vegetables, or meat, or something.”
**THEME: Needs on How to Carry Out**	**10 (91%)**	**1 (9%)**	
Multi-disciplinary	5 (100%)	0 (0%)	“If various professionals could come together—physician, nurse, maybe a dietician—and they work together to send you here or there, that would be the best solution.”
Psych	5 (83%)	1 (17%)	“That’s what I was going for, it’s that psychological help. There are some people, like myself, who are weak in many aspects and we need someone to support us through this, to tell us what we need to do, to help us make the effort and work hard, give us some examples….I’d like for someone with a background in psychology to work together with my PCP, a psychologist with knowledge of nutrition, endocrinology, but primarily focused on offering psychological assistance to the person, to tell him/her that you have a wedding to go to this Friday and, even though you have that celebration, you need to keep the doors shut. I think that’s what we’re missing. We’re weak.”
**THEME: Reinforcement / Punishment**	**10 (71%)**	**4 (29%)**	
Reinforcement– punishment	6 (60%)	4 (40%)	“But I don’t know why we have to wait for the physician to tell us. Going to the doctor means he’ll tell me that I have to stop eating this or doing that.”
Free	4 (100%)	0 (0%)	“I’ve dieted with the help of the local health center. The doctor sent me to the nurse, and the nurse simply told me that this is a 1000-calorie diet and to come back every two weeks so she could weigh me. So it’s OK, but, I think when you pay, when you go somewhere (e.g., the parapharmacy) and pay someone, I think you take it more seriously. I don’t know if it’s the money that moves us to action, but that’s the way it is. I’ve always lost weight by paying for it (the advice).”
**THEME: Outcomes**	**9 (25%)**	**27 (75%)**	
Satisfied	2 (18%)	9 (82%)	“I’m really happy with my PCP and nurse because they take care of me. They are the ones who opened my eyes and helped me see that, from the first day, that the plan was reasonable and you have to do this, that, and the other… changing food habits is more difficult for me (than stopping smoking or exercising). It’s really difficult. But, little by little, I’ve lost a lot of weight.
Behavior change	0 (0%)	6 (100%)	“The next day I called the local health center and asked for an appointment with (PCP nurse). And when I got on the scale, I almost had a fit −109 kg. “What?” I said. “I can’t believe I weight that much!” She didn’t put me on a restrictive diet. She saw me each month. I made radical changes to what I eat and my lifestyle. If I hadn’t, I know I wouldn’t be alive 10 years from now.”
Results obtained	3 (20%)	12 (80%)	“My first impression wasn’t a good one. I thought that I was going to do this (follow advice) for two or three days, or one month. And then you’ll see how I don’t lose weight. And then I lost 7 kg in the first month and I thought “Wow! This really works!”. He (My PCP) didn’t limit himself to doing the handout thing the other nurse did.”
Abandonment	4 (100%)	0 (0%)	“Later they (primary care professionals) give you a sheet with what you should and shouldn’t eat. And it seems that you’ll die of hunger, so you don’t come back the next day.”

## References

[1] World Health Organization (2018). Noncommunicable Diseases Country Profiles 2018.

[2] World Health Organization (2021). Non Communicable Diseases.

[3] United Nations THE 17 GOALS | Sustainable Development; Department of Economic and Social Affairs. https://sdgs.un.org/goals.

[4] World Health Organization (2020). Noncommunicable Diseases.

[5] World Health Organization (2004). Global Strategy on Diet, Physical Activity and Health.

[6] Hooper L. (2007). Primary prevention of CVD: Diet and weight loss. BMJ Clin. EEvid..

[7] Hartley L., Igbinedion E., Holmes J., Flowers N., Thorogood M., Clarke A., Stranges S., Hooper L., Rees K. (2013). Increased consumption of fruit and vegetables for the primary prevention of cardiovascular diseases. Cochrane Database Syst. Rev..

[8] EFSA (European Food Safety Authority) (2017). Panel on Dietetic Products Nutrition & Allergies. Dietary Reference Values for Nutrients. Summ. Rep. EFSA Support. Publ..

[9] Berry S.E., Valdes A.M., Drew D.A., Asnicar F., Mazidi M., Wolf J., Capdevila J., Hadjigeorgiou G., Davies R., Al Khatib H. (2020). Human postprandial responses to food and potential for precision nutrition. Nat. Med..

[10] Ordovas J.M., Ferguson L.R., Tai E.S., Mathers J.C. (2018). Personalised nutrition and health. BMJ.

[11] Adams S.H., Anthony J.C., Carvajal R., Chae L., Khoo C.S.H., Latulippe E.M., Matusheski N., McClung H.L., Rozga M., Schmid C.H. (2019). Perspective: Guiding Principles for the Implementation of Personalized Nutrition Approaches That Benefit Health and Function. Adv. Nutr..

[12] Jinnette R., Narita A., Manning B., McNaughton A.S., Mathers J.C., Livingstone K.M. (2021). Does Personalized Nutrition Advice Improve Dietary Intake in Healthy Adults? A Systematic Review of Randomized Controlled Trials. Adv. Nutr..

[13] Brunner E., Rees K., Ward K., Burke M., Thorogood M., Brunner E.J. (2007). Dietary advice for reducing cardiovascular risk. Cochrane Database Syst. Rev..

[14] Rees K., Dyakova M., Wilson N., Ward K., Thorogood M., Brunner E. (2013). Dietary advice for reducing cardiovascular risk. Cochrane Database Syst. Rev..

[15] Bauer E.U., Briss P.A., Goodman R.A., Bowman B.A. (2014). Prevention of chronic disease in the 21st century: Elimination of the leading preventable causes of premature death and disability in the USA. Lancet.

[16] Berman A.H., Kolaas K., Petersén E., Bendtsen P., Hedman E., Linderoth C., Müssener U., Sinadinovic K., Spak F., Gremyr I. (2018). Clinician experiences of healthy lifestyle promotion and perceptions of digital interventions as complementary tools for lifestyle behavior change in primary care. BMC Fam. Pract..

[17] Celis-Morales C., Lara J., Mathers J.C. (2015). Personalising nutritional guidance for more effective behaviour change. Proc. Nutr. Soc..

[18] World Health Organization (2017). Tackling NCDs: Best Buys and Other Recommended Interventions for the Prevention and Control of Noncommunicable Diseases.

[19] Kruk M.E., Nigenda G., Knaul F.M. (2015). Redesigning Primary Care to Tackle the Global Epidemic of Noncommunicable Disease. Am. J. Public Health.

[20] Ebrahim S., Taylor F., Ward K., Beswick A., Burke M., Smith G.D. (2011). Multiple risk factor interventions for primary prevention of coronary heart disease. Cochrane Database Syst. Rev..

[21] Alageel S., Gulliford M., McDermott L., Wright A.J. (2017). Multiple health behaviour change interventions for primary prevention of cardiovascular disease in primary care: Systematic review and meta-analysis. BMJ Open.

[22] Rubio-Valera M., Pons-Vigués M., Andres M.M., Moreno-Peral P., Berenguera A., Fernandez A. (2014). Barriers and Facilitators for the Implementation of Primary Prevention and Health Promotion Activities in Primary Care: A Synthesis through Meta-Ethnography. PLoS ONE.

[23] Elwell L., Povey R., Grogan S., Allen C., Prestwich A. (2013). Patients’ and practitioners’ views on health behaviour change: A qualitative study. Psychol. Health.

[24] Grandes G., Sanchez A., Sanchez-Pinilla R.O., Torcal J., Montoya I., Lizarraga K., Serra J. (2009). Effectiveness of Physical Activity Advice and Prescription by Physicians in Routine Primary CareA Cluster Randomized Trial. Arch. Intern. Med..

[25] Sanchez A., Grandes G., Cortada J.M., Pombo H., Martinez C., Corrales M.H., De La Peña E., Mugica J., Gorostiza E. (2017). PVS Group Feasibility of an implementation strategy for the integration of health promotion in routine primary care: A quantitative process evaluation. BMC Fam. Pract..

[26] Goldstein M.G., DePue J., Kazura A., Niaura R., Shumaker S.A., Schron E.B., Ockene J.K., McBee W.L. (1998). Models for provider–patient interaction: Applications to health behavior change. The Handbook of Health Behavior Change.

[27] Whitlock E.P. (2002). Evaluating primary care behavioral counseling interventions: An evidence-based approach. Am. J. Prev. Med..

[28] Grandes G., Sanchez A., Cortada J.M., Pombo H., Martinez C., Balagué L., Corrales M.H., De La Peña E., Mugica J., on behalf of the PVS group (2017). Collaborative modeling of an implementation strategy: A case study to integrate health promotion in primary and community care. BMC Res. Notes.

[29] Damschroder L.J., Aron D.C., Keith E.R., Kirsh S.R., A. Alexander J., Lowery J.C. (2009). Fostering implementation of health services research findings into practice: A consolidated framework for advancing implementation science. Implement. Sci..

[30] Martinez C., Bacigalupe G., Cortada J.M., Grandes G., Sanchez A., Pombo H., Bully P. (2017). PVS Group The implementation of health promotion in primary and community care: A qualitative analysis of the ’Prescribe Vida Saludable’ strategy. BMC Fam. Pract..

[31] Cane J., O’Connor D., Michie S. (2012). Validation of the theoretical domains framework for use in behaviour change and implementation research. Implement. Sci..

[32] Saldaña J. (2021). The Coding Manual for Qualitative Researchers.

[33] Brunner-Sperdin A., Peters M. (2009). What influences guests’ emotions? The case of high-quality hotels. Int. J. Tour. Res..

[34] Braun V., Clarke V. (2014). What can “Thematic Analysis” Offer Health and Wellbeing Researchers?. Int. J. Qual. Stud. Health Well-being.

[35] Grandes G., Sanchez A., Cortada J.M., Balague L., Calderon C., Arrazola A., Vergara I., Millan E. (2008). Is integration of healthy lifestyle promotion into primary care feasible? Discussion and consensus sessions between clinicians and researchers. BMC Health Serv. Res..

[36] Bully P., Sanchez A., del Olmo E.Z., Pombo H., Grandes G. (2015). Evidence from interventions based on theoretical models for lifestyle modification (physical activity, diet, alcohol and tobacco use) in primary care settings: A systematic review. Prev. Med..

[37] Alageel S., Gulliford M.C., McDermott L., Wright A.J. (2018). Implementing multiple health behaviour change interventions for cardiovascular risk reduction in primary care: A qualitative study. BMC Fam. Pract..

[38] Damschroder L.J., Lowery J.C. (2013). Evaluation of a large-scale weight management program using the consolidated framework for implementation research (CFIR). Implement. Sci..

[39] Brotons C., Björkelund C., Bulc M., Ciurana R., Godycki-Cwirko M., Jurgova E., Kloppe P., Lionis C., Mierzecki A., Piñeiro R. (2005). Prevention and health promotion in clinical practice: The views of general practitioners in Europe. Prev. Med..

[40] Peckham S., Hann A., Kendall S., Gillam S. (2017). Health promotion and disease prevention in general practice and primary care: A scoping study. Prim. Health Care Res. Dev..

[41] Fuller T.L., Backett-Milburn K., Hopton J.L. (2003). Healthy eating: The views of general practitioners and patients in Scotland. Am. J. Clin. Nutr..

[42] Grandes G., Sanchez A., Torcal J., Sánchez-Pinilla R.O., Lizarraga K., Serra J. (2008). Targeting physical activity promotion in general practice: Characteristics of inactive patients and willingness to change. BMC Public Health.

[43] Grandes G., Sanchez A., Montoya I., Sanchez-Pinilla R.O., Torcal J., on behalf of the PEPAF Group (2011). Two-Year Longitudinal Analysis of a Cluster Randomized Trial of Physical Activity Promotion by General Practitioners. PLoS ONE.

[44] Sanchez A., Bully P., Martinez C., Grandes G. (2015). Effectiveness of physical activity promotion interventions in primary care: A review of reviews. Prev. Med..

[45] Damschoder L.J., Goodrich E.D., Robinson C.H., E. Fletcher C., Lowery J.C. (2011). A systematic exploration of differences in contextual factors related to implementing the MOVE! weight management program in VA: A mixed methods study. BMC Health Serv. Res..

[46] Walseth L.T., Abildsnes E., Schei E. (2011). Patients’ experiences with lifestyle counselling in general practice: A qualitative study. Scand. J. Prim. Health Care.

[47] Mazza D., Shand L., Warren N., Keleher H., Browning C., Bruce E.J. (2011). General practice and preventive health care: A view through the eyes of community members. Med. J. Aust..

[48] Celis-Morales C., Livingstone K., Rocha F.P., Navas-Carretero S., San-Cristobal R., O’Donovan C.B., Moschonis G., Manios Y., Traczyk I., Drevon C.A. (2019). Frequent Nutritional Feedback, Personalized Advice, and Behavioral Changes: Findings from the European Food4Me Internet-Based RCT. Am. J. Prev. Med..

[49] Moreno-Peral P., Cerón S.C., Fernández A., Berenguera A., Andres M.M., Pons-Vigués M., Motrico E., Rodríguez-Martín B., Bellón J.A., Rubio-Valera M. (2015). Primary Care Patients’ Perspectives of Barriers and Enablers of Primary Prevention and Health Promotion—A Meta-Ethnographic Synthesis. PLoS ONE.

